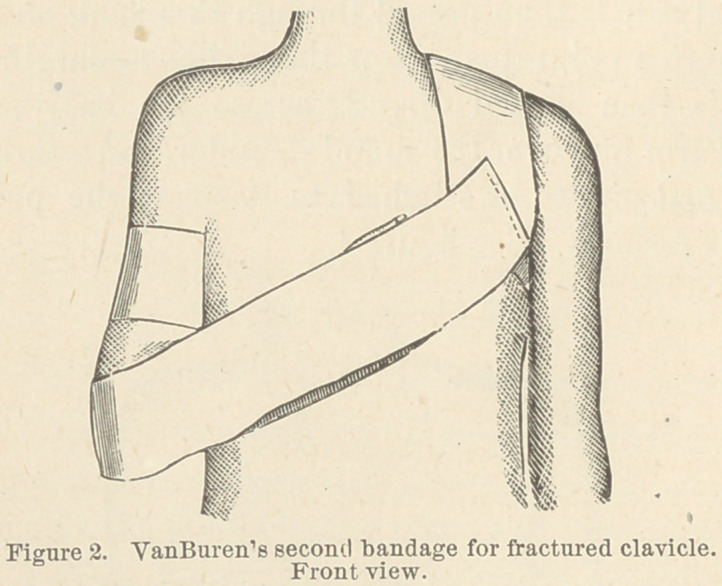# A New Method of Treating Fracture of the Clavicle

**Published:** 1877-09

**Authors:** Henry Van Buren

**Affiliations:** Chicago


### A NEW METHOD OF TREATING FRACTURE OF THE CLAVICLE.

Henry Van Buren, M. D., Chicago.

While one of the visiting physicians of the Central Free Dispensary about two years ago, I treated a patient for fracture of the clavicle, adopting the plan of my friend Dr. Lewis A. Sayre, of New York, using two strips of adhesive plaster without any axillary pad.

I became convinced at once, that the principle advocated by Prof. Sayre, was undoubtedly the correct one; but before I had gone very far in the use of the adhesive strips, I fouud that my patient, a young native of Ireland, began to tear them off. The weather was warm, and, to use the language of the lad, they "itched him."

Finding this difficulty in holding the arm and shoulder back by a hitch around the body with adhesive plaster, the thought struck me, that I would make a hitching post of the sound shoulder instead. Not as in the old plan of a figure of eight around both shoulders, but upon that which I will now lay before my brethren in the profession.

To make known my plan in a sentence—I make attachment to the middle of the arm on the fractured side; draw the arm backward until the clavicular portion of the pectoralis major muscle is put sufficiently on the stretch to overcome the sterno-cleido-mastoid, and then make a hitching post of the sound shoulder to hold these muscles in extension, and by this extension with the sling, which will be hereafter described, the ends of the fractured clavicle are held in apposition.

I make the first bandage 3 or 4 inches wide out of unbleached cotton, of double thickness and sufficient length. On one end of this bandage a loop is made, by returning the bandage on itself, and fastening the end with a few stitches. The hand on the injured side is then passed through this loop, and the loop carried up to a point just below the axillary margin. This bandage is then passed directly across the back, and under the sound arm and over the sound shoulder, and returned across the back, and pinned or stitched to itself at the point where the loop is formed. See figure 1.

Figure 1. VanBuren's first bandage for fractured clavicle. Back view.

The second bandage is then made and applied as follows: I flex the arm of the injured side, and place the hand on the chest, pointing in the direction of the sound shoulder; I then take a piece of the same material as used in the first instance, and make a bandage 4 inches wide, of double thickness and sufficient length, and pin or stitch one end of this bandage to the lower margin of the first bandage, in front of the sound shoulder. It is then passed diagonally downward, and across the chest under the hand and forearm which has been flexed upon the chest, and carried around the arm at the elbow, and back on the dorsal surface of the forearm and hand to the point from which it started, and this end also pinned to the first bandage.

I then stitch the lower margins of this bandage together for a distance of about three inches at the elbow, thus forming a trough for the elbow to rest in. I also do the same at the upper end of this bandage, which forms another short trough for the hand to rest in. See figure 2.

Figure 2. VanBuren's second bandage for fractured clavicle. Front view.

This bandage or sling may be made as described above, before it is applied, and the elbow placed in the lower trough and the hand in the upper one; and the upper ends of the bandage pinned to the lower margin of the first bandage, at a point opposite the sound shoulder, as above indicated; indeed I prefer this plan because more convenient.

This sling serves the triple purpose of drawing the lower end of the arm forward and upward, and thus throwing the injured shoulder backward. It supports the fore-arm and hand in a comfortable and quiet position, and last, it prevents the first bandage from cording under the sound arm by its attachment to its lower margin.

To prevent the first bandage from producing excoriation in the axilla of the sound side, I usually cushion the bandage at this point by stitching on two or three extra thicknesses of the cotton cloth. The same may be done at the loop,—around the arm of the injured side, if necessary.

What is presented, then, for the consideration of the profession in this method is—

1st. The great simplicity of the appliance.

2d. The complete retention of the fragments in apposition.

3d. The comparative ease with which the bandage is worn.

The deformity which takes place in fracture of the clavicle is too well known to require any description; viz., that the shoulder falls downward, forward and inward, and that the outer end of the sternal fragment overlaps the inner end of the acromial portion of the clavicle.

The indications to be fulfilled in the treatment are also well known; viz., to draw the shoulder upward, outward and backward, and retain it there, and thus by virtue of this position, hold the fractured ends in apposition.

It will be observed that the first bandage, as presented in Fig. 1, not only draws the shoulder backward, but has a lifting tendency, the bandage being at a higher point, where it passes over the sound shoulder than where attached to the arm on the injured side, hence the shoulder is drawn upward; also that the deltoid and biceps muscles are quieted by the loop around the arm.

Let the surgeon himself stand erect and thrust backward and upward his own shoulder, the one supposed to be the injured one, and flex the fore-arm upon the chest, with the hand pointing in the direction of the sound shoulder, and he has at once secured the position and fulfilled all the indications desired in fracture of the clavicle; and the bandages presented in this paper retain this position in a very simple and practical manner.

A patient of mine under treatment for this injury, was brought before the Chicago Medical Society, at one of its regular meetings in May last, after union had taken place;—and I think the gentlemen who were present can say that there was little or no deformity in the case before them.

I also had the privilege of doing what was so much desired before submitting this paper for publication, that of bringing this method before a number of surgeons of high standing in the profession, at the late meeting of the American Medical Association, among whom were Dr. Lewis A. Sayre, of New York; Drs. Gunn and Powell, of Chicago; and Drs. Bridge and Hyde, associate editors of the MEDICAL JOURNAL AND EXAMINER, who approved of the plan laid before them.

I was eager for the opinion of Prof. Sayre, who was the first to put into practice the principle laid down in this method, and the plan received his hearty approval.

I have treated every case of fractured clavicle upon this plan, which I have been called upon to attend for the past two years, modifying the appliance from time to time, until the indications sought after were more perfectly acquired.

At the beginning of the third week, or earlier, the bandages should be removed occasionally, and passive motion of the elbow and shoulder made.

We are of the opinion that judicious movement of all fixed joints has been too long delayed, by most surgeons in cases of fracture. In the fracture presented in this paper, with the bandages used, early movement is indispensable, inasmuch as the parts are held so completely quiet.

And now, if any apology is needed for trying to present a new way for treating an old fracture, the excuse must be found in the fact, that we think the old plans were failures, notwithstanding the many and complicated means devised to secure retention.

Dr. Sayre has quoted, in his pamphlet on this fracture, from a dozen authors, running back to the days of Hippocrates, showing that this injury has always been attended with deformity.

In Prof. Hamilton's work on Fractures and Dislocations, the author quotes from fifty-seven different authors, to sustain his own observations, that this fracture is nearly always followed by deformity.

Miller, Ferguson, Simpson, Hancock, South, and many others of England, and a grand array in other countries, have all had their wedged-shaped pads, and never-ending turns of the bandage around the body, but I cannot see that they accomplished more than to keep the fracture quiet, and thus facilitate a kind of union with, as they all acknowledged, more or less deformity.

South says that he does not like any apparatus which draws the shoulders backward. If the author means both shoulders, we are agreed; but I want one shoulder, and that the injured one, drawn backward and well backward at that,—for herein we get extension and counter extension too, if you please, the thing so essential in fractures of all long bones, and we cannot get this in any other way. The pad under the arm does not cause adequate extension, nor will it ever do so, no matter how large or in what manner placed.

The figure of 8 bandage of modern use, is to me exceedingly objectionable, for one important reason, if for no other. If the fracture is in the middle third of the clavicle, or near the middle at all, the bandage presses down over the site of injury, and particularly over the inner end of the outer fragment, the very end already dragged down by the weight of the shoulder, and just here is one of the valuable points in what we have termed a new method. The injured shoulder is entirely free from any depressing or other bandage.. I do not even allow the patient to wear a suspender over the injured shoulder.

DeSault, Dupuytren, Cloquet, Salamon, and Jæger, all carry their bandages over the injured shoulder, and all admit, as they must do, that they get angular deformity.

The "postural position" might do quite well for an indolent man, but even then we might fail in getting union again. This is an age of fresh air and hygiene, and every patient, as far as practicable, should have the advantages of out-door exercise.

I am no stickler for any particular apparatus in the treatment of fracture, any more than I would be for any particular medicine in disease. Whatever accomplishes the end in the most simple manner under existing circumstances, is generally, if not always, the best, and the plan for treating fracture of the clavicle, as presented in this paper, is in keeping with this doctrine, and is brought before the profession with confidence, and we believe that a good result can be attained in the hands of any surgeon, if the method is faithfully and intelligently carried out.

Chicago, August 1st, 1877.

## Figures and Tables

**Figure 1. f1:**
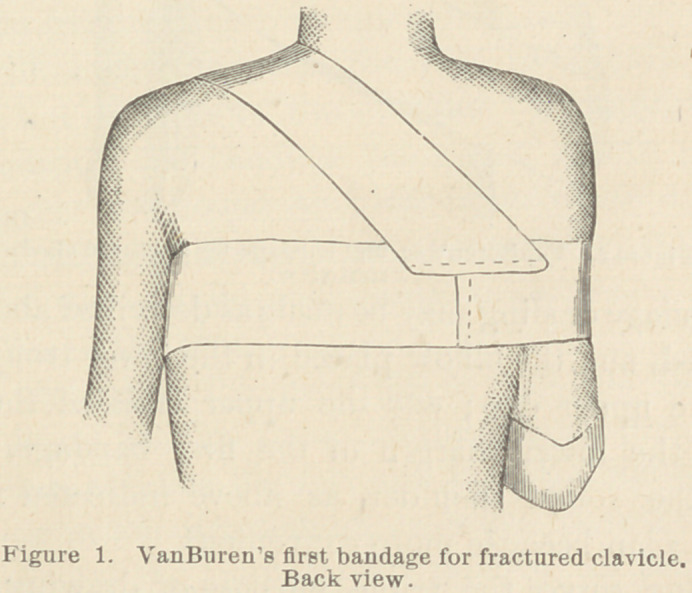


**Figure 2. f2:**